# Pachy-meningitis Interna—Reticulated Round Cell Sarconia of Dura-Mater and Exostosis of the Inner Table of the Skull

**Published:** 1881-10

**Authors:** Geo. M. Parkinson

**Affiliations:** Middletown, Conn.


### ART. XIX.—PACHY-MENINGITIS INTERNA.—RETICULATED ROUND CELL SARCONIA OF DURA-MATER AND EXOSTOSIS OF THE INNER TABLE OF THE SKULL.

On August 17, 1881, I was called about forty miles from my place of business to see the horse from which I obtained the following history. The animal was a bay gelding between fifteen and sixteen hands high, aged about fifteen years; it was in fine condition, and weighed between eleven and twelve hundred pounds. The horse had previously worked on a fire steame outlived two mates. About three years ago the present owner bought him from a regular horse-dealer to work on his farm, and the horse proved to be a true, faithful and useful worker. Last fall the animal had an attack of nasal catarrh, and was treated for the same by a veterinary surgeon. Since then there has been a bulging outward over the frontal sinuses. After the above attack, the owner informed me that the animal "had never been like himself."

During the last two weeks of his life he became very lazy, and seemed not to care which way or how he went. One evening the owner turned him out to pick some grass and he ran away, but came back home again in the morning. At another time he deliberately went over a stone wall, apparently without any knowledge of the wall being in his way.

When I first saw the animal the symptoms were as follows: The horse was very uneasy and kept continually walking around the stable. Every few minutes he would press the anterior portion of the neck on the left side against the partition, often so hard, at the same time turning his body so far to the right, that he nearly strangled himself. The temperature was only a trifle above the normal; pulse from 44 to 48; respiration did not seem to be disturbed. He ate but little. Food was often taken into the mouth and held for some time, when he would get a portion well back into the mouth and commence grinding, but swallowed very little, if any. When given a pail of water to drink from, he would plunge his nose well into the pail and blow the water all around, but drank scarcely any. The day on which he died, however, he drank a whole pail of water, and succeeded in eating and swallowing a few corn leaves and a half dozen pears. Five hours before death he became wild and frantic, breaking everything that came in his wav, and it was with difficulty that he could be kept walled in. About one hour before death, however, he dropped down and lay quiet, but snored heavily until death, which occurred without a struggle.

Before I saw the animal the parties had been administering bromide of potassium. I ordered a fluid diet, with a little fresh grass; also,

℞—Pulv. aloes, barb., ℥ x.

Hydrag. chloridi, mite, gr. xx.

Misce; et fiat in bolus.

Sig.; to be administered as soon as procured.

The bromide salt was continued in one and two-drachm doses, also enemas. On Saturday previous to his death, the bromide salt was discontinued, and chloral hydrate, in half-ounce doses, three times a day, substituted, also:

℞—Pulv. aloes, Barb., ℥ iij.

Pulv. zingiber, rad., q. s., ad.

Misce; et fiat in bolus.

Sig.; p. r. n.

Tinctura aconitii, in minute doses, was given every two hours.

During the evening he became wild, and I injected morphine hypodermically whenever an opportunity offered. All treatment, however, availed nothing, and the unfortunate animal died at 10 o'clock A. M., August 21st, 1881.

A necropsy was made ten hours after death. The brain weighed twenty-four ounces. There was a large depression upon the upper surface of the anterior lobe of the left hemisphere. Directly over this depression, between the dura and pia mater, there was a deep layer of thick, yellowish, semi-fluid matter. The accumulation had pressed the pia away from the dura mater. There was a bone-like tumor growing from the internal table of the cranium. This bony outgrowth was so situated that it pressed into the side of the cerebellum of the left side. The vessels of the membranes were distended with blood. The frontal sinuses were filled with a fatty gelatinous looking substance (fibrin). The lateral sinuses were also partially filled with a similar looking substance, but a little more flaky in character. The walls of the sinuses were somewhat thickened. Growing from the inner surface of the dura-mater there was a small neoplasm, about three-quarters of an inch in diameter.

The brain, the portion of dura-mater to which the tumor was attached, and a portion of the exostosis, was sent to the School of Histology in connection with the Columbia Veterinary College, for examination. The following report was rendered by Dr. Porter:

"The brain substance proper appeared to be normal. The depressions on the surface, which were so marked at the necropsy, were not as perceptible when received. The portion of the bony outgrowth was found to be true bone, and undoubtedly an exostosis. When a transverse section of the tumor was made, the central portion was much lighter in color than the outer part.

"*Microscopical examination of tumor:*—The general structure was a reticulated network, containing large and small round connective tissue corpuscles. The cells were surrounded by a delicate intercellular substance. Portions of the growth were also very vascular, and filled with thin walled capillaries, which were distended with blood corpuscles."

GEO. M. PARKINSON, D.V.S.

MIDDLETOWN, Conn.

